# Mechanism of protonophores-mediated induction of heat-shock response in *Escherichia coli*

**DOI:** 10.1186/1471-2180-9-20

**Published:** 2009-01-29

**Authors:** Bimal Jana, Subrata Panja, Swati Saha, Tarakdas Basu

**Affiliations:** 1 Department of Biochemistry and Biophysics, University of Kalyani, Kalyani – 741 235, West Bengal, India

## Abstract

**Background:**

Protonophores are the agents that dissipate the proton-motive-force (PMF) across *E. coli *plasma membrane. As the PMF is known to be an energy source for the translocation of membrane and periplasmic proteins after their initial syntheses in cell cytoplasm, protonophores therefore inhibit the translocation phenomenon. In addition, protonophores also induce heat-shock-like stress response in *E. coli *cell. In this study, our motivation was to investigate that how the protonophores-mediated phenomena like inhibition of protein translocation and induction of heat-shock proteins in *E. coli *were correlated.

**Results:**

Induction of heat-shock-like response in *E. coli *attained the maximum level after about 20 minutes of cell growth in the presence of a protonophore like carbonyl cyanide m-chloro phenylhydrazone (CCCP) or 2, 4-dinitrophenol (DNP). With induction, cellular level of the heat-shock regulator protein sigma-32 also increased. The increase in sigma-32 level was resulted solely from its stabilization, not from its increased synthesis. On the other hand, the protonophores inhibited the translocation of the periplasmic protein alkaline phosphatase (AP), resulting its accumulation in cell cytosol partly in aggregated and partly in dispersed form. On further cell growth, after withdrawal of the protonophores, the previously accumulated AP could not be translocated out; instead the AP-aggregate had been degraded perhaps by an induced heat-shock protease ClpP. Moreover, the non-translocated AP formed binary complex with the induced heat-shock chaperone DnaK and the excess cellular concentration of DnaK disallowed the induction of heat-shock response by the protonophores.

**Conclusion:**

Our experimental results suggested that the protonophores-mediated accumulation and aggregation of membrane proteins (like AP) in cell cytosol had signaled the induction of heat-shock proteins in *E. coli *and the non-translocated protein aggregates were possibly degraded by an induced heat-shock protease ClpP. Moreover, the induction of heat-shock response occurred by the stabilization of sigma-32. As, normally the DnaK-bound sigma-32 was known to be degraded by the heat-shock protease FtsH, our experimental results further suggested that the engagement of DnaK with the non-translocated proteins (like AP) had made the sigma-32 free and stable.

## Background

The heat-shock response is a universal reaction in nature to defend cells against the temperature-induced damage. Cells of bacteria or almost any organism respond to sudden increase in temperature by synthesizing a set of proteins called the heat-shock proteins (hsps). In *E. coli*, heat-shock regulon includes genes for about 30 proteins and is induced after a temperature up-shift from 30 to 45°C. The hsps counter the effects of heat by serving as 1) molecular chaperones (e.g., GroEL, GroES, DnaK, DnaJ, ClpB etc.) that assist in the refolding of the partially denatured proteins and 2) proteases (e.g., Lon, ClpP, FtsH etc.) that degrade and remove the permanently denatured proteins [1]. Not only important during heat stress, many hsps are present at the basal level in cells to assist protein folding [2]. Transcription of the heat shock genes is known to be initiated by RNA polymerase, which contains the alternative sigma factor sigma-32 [3]. At normal growth condition, cellular concentration of sigma-32 is very low (10–30 copies/cell at 30°C) and increases up to 12–15 folds with the temperature up-shift [4].

Instead of heat, cytoplasmic accumulation of the membrane or periplasmic proteins elevates the syntheses of hsps in *E. coli*. Any membrane or periplasmic protein of *E. coli *is known to be synthesized initially in cell cytoplasm as precursor form, which contains an N-terminal signal-sequence [5]. The signal sequence targets the precursor towards the plasma membrane translocase that transports the precursor across the membrane [6]. The signal peptide is then cleaved by a signal peptidase, an integral membrane protein with active site facing the periplasm [7]. The matured protein is then positioned at its membrane or periplasmic location with functionally correct orientation. The PMF across *E. coli *plasma membrane acts as an energy source for protein translocation [8,9]. The inhibition of translocation and consequent storage of membrane proteins in cell cytosol is found to induce hsps in export deficient mutants (where the multi-subunit translocase is nonfunctional) [10,11], in signal sequence mutants (where the precursor proteins cannot be targeted to the translocase) [12,13], and in wild type cells treated with protonophores like CCCP or DNP [14,15]. However, it is still obscure how the inhibition of protein translocation phenomenon is related to the induction of cellular heat-shock response at the molecular level. Therefore, in the present study, we target to investigate 1) how the cellular level of the heat-shock regulator protein sigma-32 is modulated under the condition of inhibition of protein translocation by the protonophores like CCCP/DNP, 2) what is the final fate of the non-translocated proteins, stored in cell cytoplasm and 3) how the induced hsps do interact with the non-translocated proteins.

## Methods

### Bacterial strains and plasmid

The *E. coli *strain Mph42 [16], mostly used in this study, was a generous gift from Dr. Jonathan Beckwith, Department of Microbiology and Molecular Genetics, Harvard Medical School, Boston, USA. The *E. coli *strains JT4000 (∇ *lon-510*) [17] and SG22159 (*clpP:: kan*) [17], mutants of the Lon and ClpP protease respectively, and their wild type strain SG20250 (*MC4100, clp*^+^, *lon*^+^) [17] were kindly gifted by Dr. Susan Gottesman, Laboratory of Molecular Biology, NCI, NIH Bethesda, USA. Sigma-32 was isolated from *E. coli *strain BB2012 (a His-tagged clone), a kind gift from Dr. Matthias P. Mayer, Institute for Biochemistry and Molecular Biology, University of Freidburg, Germany. The plasmid pET vector containing *dnaK *gene was obtained from Prof. C. K. Dasgupta, Department of Biophysics, Molecular Biology & Genetics, University of Calcutta, Kolkata, India.

### Media, Reagents and Chemicals

All the components of growth medium, the required antibiotics, sucrose, lysozyme, NONIDET-P40 (NP40) and the electrophoresis reagents were purchased from Pharmacia Biotech., Sweden; purified *E. coli *AP, DNP, CCCP, antibody to GroEL, 4-chloro-1-napthol and Freunds adjuvant from Sigma-Aldrich, USA; Ni-NTA Agarose from QIAGEN, Germany; HRP-conjugated goat anti-rabbit I_g_G (secondary antibody) and proteinA-CL agarose from Genei, India; the Nitrocellulose transfer membrane from BioRad Laboratories, USA; ^35^S-methionine from Board of Radiation and Isotope Technology, India; H_2_O_2_, Tween-20 and anti-DnaK antibody from Merck, India; Isopropyl β-D-thiogalacto pyranoside (IPTG) and p-nitrophenyl phosphate (PNPP) from Sisco Research Laboratories, India.

### Western blot experiment

This experiment was performed according to the method described in [13]. Interested specific protein on the blotted membrane was identified by using the antiserum of the protein (raised in rabbit) as the primary antibody, HRP-conjugated goat anti-rabbit I_g_G as the secondary antibody and 4-chloro-1-napthol and H_2_O_2 _as the HRP substrates.

### Pulse-label/Pulse-chase and immunoprecipitation experiments

Cells of *E. coli *Mph42 were initially grown to the log phase (up to [OD]_600 nm _≈ 0.3, i.e., 1.5 × 10^8 ^cells/ml) at 30°C in MOPS medium (where the methionine concentration was 1/10th of the normal MOPS medium [18]) and were subsequently transferred to the methionine-free MOPS medium.

For pulse-label and immunoprecipitation experiment, log phase grown cells in methionine-free MOPS medium were allowed to grow further at 30°C. At different instants of growth, 1 ml cell aliquot was withdrawn to label with ^35^S-methionine (100 μCi/ml) for 1 min. The labeled cells were treated with 5% Trichloroacitic acid. The protein precipitate was washed with 80% cold acetone. The air dried precipitate was suspended in 50 μl of 50 mM Tris buffer (pH 8.0) containing 1% SDS and 1 mM EDTA. It was then heated at 100°C for 3 min; 30 μl of this sample was diluted with 1 ml of Triton X-100 buffer [2% Triton X-100, 50 mM Tris, pH 8.0, 150 mM NaCl and 1 mM EDTA] and centrifuged to remove nonspecific precipitates. From the supernatant, for immunoprecipitation of any protein, requisite amount of the antibody to that protein was added and subsequently incubated overnight at 0°C. To this incubated sample, 50 μl of proteinA-CL agarose was added and incubated further at 0°C for 20 min. The immunocomplex was washed and finally suspended in 50 μl of 2× sample buffer [19], heated at 100°C for 3 min prior to loading on 12% SDS-polyacrylamide gel for electrophoresis; finally phosphorimaging of the gel was performed in Typhoon 9210 (GE Health Care).

For pulse-chase and immunoprecipitation experiment, log phase grown cells in methionine-free MOPS medium were radio-labeled with ^35^S-methionine (at a concentration of 30 μCi/ml of cell culture) for the required time and the label was subsequently chased by 0.2 M cold methionine. At different instants of chasing, cell aliquot was withdrawn to extract proteins by the method of Oliver and Beckwith [19]. Subsequent steps of immunoprecipitation from the cell extract with requisite amount of an antibody, gel electrophoresis and phosphorimaging were done as described above.

### Induction, activity assay and determination of location of AP

For the induction of AP, *E. coli *MPh42 cells were grown in the phosphate-less MOPS medium at 30°C, as described in [13]. At different instants of induction, an aliquot of 1.0 ml cell suspension was collected over 0.2 ml toluene and the activity of AP was assayed as described in [13], using PNPP as the substrate. The amount of AP, which led to a change of absorbance of p-nitrophenol by 0.1 per 6 min of enzyme-substrate reaction, had been considered as one unit of the enzyme [13].

For determination of the location of AP, the periplasmic, cytoplasmic and membrane fractions of cells were isolated from 1.0 ml of AP induced cell culture, as described in [20]. After electrophoresis of the fractions in 12% SDS-polyacrylamide gel, 'western blot' experiment with anti-AP antibody was performed.

### Isolation of aggregated proteins

Isolation of total soluble (containing dispersed protein pool) and insoluble (containing aggregated protein pool) cell fractions was based on the method described in [21]. Cells were allowed to grow at 30°C in MOPS medium up to bacterial OD_600 nm _~0.5. 25.0 ml of grown culture was rapidly cooled to 0°C and centrifuged at 4°C for 10 min at 6000 rpm. The cell pellet was re-suspended in 80 μl of buffer A [10 mM potassium phosphate buffer (pH-6.5); 1.0 mM EDTA; 20% (w/v) sucrose and 1.0 mg/ml lysozyme] and incubated for 30 min on ice. To the cell suspension, 720 μl of buffer B [10 mM potassium phosphate buffer (pH-6.5); 1 mM EDTA] was added and the cells were dipped in ice to sonicate by microtip ultrasonicator (using level 2, 1 min, 50% duty, three cycles). Intact cells were removed by centrifugation at 2000 g for 15 min at 4°C. The supernatant was further centrifuged at 15000 g for 20 min at 4°C and the pellet was collected. The pellet, which contained membrane and aggregated proteins, was washed with and finally re-suspended by brief sonication in 320 μl of buffer B. 80 μl of 10% (v/v) NP40 was then added to the suspension, mixed well and centrifuged at 15000 g for 30 min at 4°C to isolate the aggregated proteins as the pellet and to remove the membrane proteins as supernatant. The steps of re-suspension in buffer B, addition of NP40 and subsequent centrifugation were repeated three times. NP40-insoluble aggregated protein pellets were washed with 400 μl buffer B and finally re-suspended in 200 μl of buffer B.

### Isolation and purification of sigma-32

The isolation and purification of the His-tagged sigma-32 from *E. coli *strain BB2012, using the Ni^2+^-NTA agarose column, were carried out according to [22].

### Immunization

The antibodies of AP and sigma-32 were raised separately according to the method of Oliver and Beckwith [19] as described in [13].

## Results and discussion

**Figure 1:**
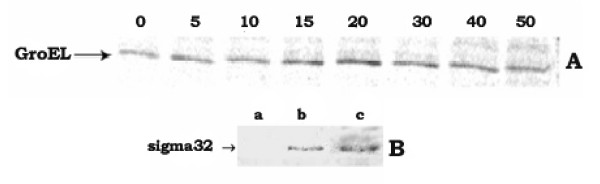
**A. *Rate of synthesis of GroEL in E. coli MPh42 cells at different instants of growth in the presence of 50 μM CCCP***. Pulse-label at 0, 5, 10, 15, 20, 30, 40 and 50 minutes of cell growth and subsequent immunoprecipitation experiment using anti-GroEL antibody was performed as described in 'Methods'. B. *The level of sigma-32 in the CCCP-treated E. coli MPh42 cells*. Log phase grown cells were divided into three parts. One part was grown at 30°C, one part was grown at 50°C and the other part was grown in the presence of 50 μM CCCP at 30°C. After 20 min of growth, 1 ml cell aliquot was withdrawn from each set. Cellular proteins were extracted by boiling the cells with SDBME buffer [18] and equal amount of protein from each extract, estimated by Bradford method [37], was electrophoresed on 12% SDS-polyacrylamide gel and subsequently the western blot study was performed using anti-sigma-32 antibody. Lane a: cells grown at 30°C; lane b: cells grown at 50°C and lane c: cells grown at 30°C in the presence of 50 μM CCCP.

In this study, investigations were carried out to establish the correlation in molecular detail between the phenomena of inhibition of protein translocation and induction of hsps in *E. coli*, grown in the presence of protonophores like CCCP and DNP. Therefore, growth of *E. coli *cells in the presence of different concentrations of the protonophores was studied first and the results indicated that the increasing concentrations of CCCP (0 – 50 μM) or DNP (0 – 1.5 mM) in the growth medium had gradually slowed down the cell growth, causing bacteriostatic condition at 50 μM CCCP or 1.5 mM DNP (data not shown). When checked using 2-D gel electrophoresis technique, cell growth in the presence of CCCP (50 μM) or DNP(1.5 mM) was found to induce the hsps like ClpB, DnaK, GroEL, GrpE, ClpP, and GroES in *E. coli *cell (results not shown); protonophores-mediated induction of hsps were reported earlier (14, 15). As, in all the following experiments, the results for CCCP (50 μM) and DNP (1.5 mM) separately were qualitatively similar, the results for the CCCP only have been presented here. At different intervals of growth in the presence of CCCP, when the rate of GroEL synthesis was investigated by the pulse-label and immunoprecipitation experiment using anti-GroEL antibody, the result showed that the rate had increased with time up to 20 min (fig. 1A), beyond which it had declined. This implied that the maximum induction of hsps had taken place after 20 minutes of cell growth in the presence of 50 μM CCCP. After 20 min of cell growth, when the western blot experiment of cell extract was performed using anti-sigma-32 antibody, the result (fig. 1B) showed that the cellular level of the heat-shock regulator protein sigma-32 had also been increased (lane c) by the CCCP treatment. Fig. 1B also showed that the level of sigma-32 in normal cells was so low in amount that it had no trace (lane a) in the western blot. Similar enhancement of cellular sigma-32 level was found to take place in cells grown at 50°C (lane b).

Under heat stress, the increase in sigma-32 was known to be caused by two means – by the increase in sigma-32 translation and by the stabilization of normally unstable sigma-32. Control of sigma-32 translation was mainly mediated by two *cis*-acting elements on sigma-32 mRNA; extensive base pairing between the elements formed secondary structure in sigma-32 mRNA, which had prevented its entry into the ribosome and consequently the translation initiation. The thermal induction of translation resulted from melting of the mRNA secondary structure at increased temperature [23]. Again, control of sigma-32 stabilization is mediated by the hsps like DnaK/J and FtsH; normally at 30°C, the DnaK/J chaperone system binds with sigma-32, limiting its binding to core RNA polymerase [24] and the FtsH, an ATP-dependent metalloprotease, degrades sigma-32 (bound with DnaK/J) [25,26]. Upon heat stress, the chaperone system DnaK/J becomes engaged with the increased cellular level of unfolded proteins and thus makes the sigma-32 free and stable [27].

**Figure 2:**
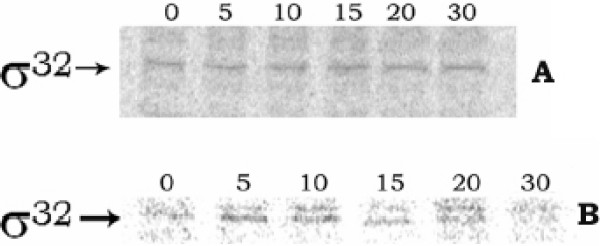
**Rate of s*ynthesis of sigma-32 at different instants of cell growth***. A and B represent the result of cell growth at 30°C in the presence of 50 μM CCCP, and at 50°C respectively. Pulse-label at 0, 5, 10, 15, 20, 30 minutes of cell growth and subsequent immunoprecipitation experiment using anti-sigma-32 antibody was performed as described in 'Methods'.

**Figure 3:**
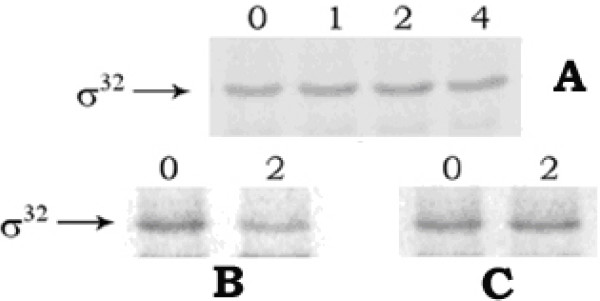
***Stability of sigma-32 in E. coli MPh42 cells***. Pulse-chase and immunoprecipitation experiment was performed using 100 μCi/ml ^35^S-methionine and anti-sigma-32 antibody, as described in 'Methods'. Panel A: cells grown at 30°C in the presence of CCCP, panel B: control cells at 30°C, and panel C: cells submitted to 50°C. The numbers in the lanes signify the time of chasing in minutes.

At different intervals of growth in the presence of CCCP, when the rate of sigma-32 synthesis was measured by the pulse-label and immunoprecipitation experiment, no change in the rate with the time of cell growth was observed (fig. 2A); whereas in cells grown at 50°C, the rate had increased up to 5 min (fig. 2B), after which it declined. Therefore, the rise in cellular sigma-32 level and thereby induction of hsps in *E. coli *by CCCP treatment did not occur by the enhanced synthesis of sigma-32. This result also indicated that the CCCP could not denature the secondary structure present in sigma-32 mRNA and thus entry of the mRNA into the ribosome and consequent increase of translation had been prevented. On the other hand, when the sigma-32 stabilization was investigated with the help of pulse-chase and immunoprecipitation experiment, no change in sigma-32 band intensity had been observed in the CCCP-treated cells up to 4 minutes of chasing (fig. 3A); whereas in case of control cells, sigma-32 intensity had been almost halved in 2 minutes of chasing (fig. 3B), signifying stabilization of sigma-32 in cells by CCCP treatment. When checked, sigma-32 was also found to be stabilized in cells grown at 50°C (fig. 3C). The above results, therefore, implied clearly that for induction of hsps in the CCCP-treated cells, cellular level of sigma-32 had been increased, not by its increased rate of synthesis, but by its increased stabilization.

**Figure 4:**
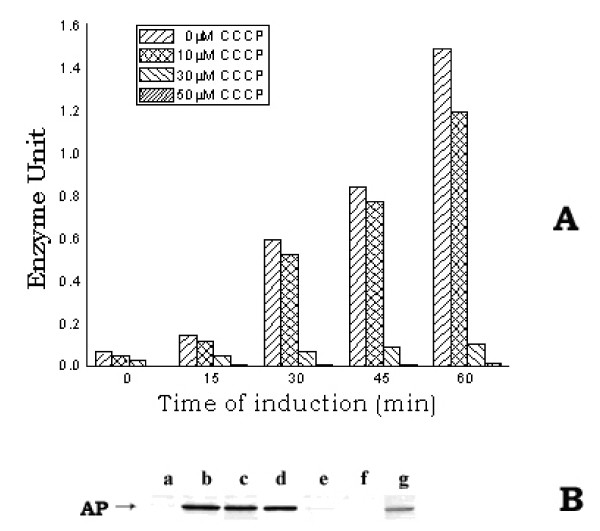
**A. L*evel of active AP in E. coli MPh42 cells grown in the presence of different concentrations of CCCP***. Cells were initially grown to log phase (~1.5 × 10^8 ^cells/ml) at 30°C in complete MOPS medium and were then transferred to phosphate-less MOPS medium. The re-suspended cells were divided in different parts to treat with the different concentrations of CCCP (0, 10, 30 and 50 μM). The divided cell cultures were then allowed to grow further at 30°C for induction of AP. At different intervals of time, a 1.0 ml cell aliquot was withdrawn from each culture to assay the active AP level. B. W*estern blot of the different fractions (periplasmic, cytoplasmic and membrane) of E. coli MPh42 cells grown in the presence of CCCP (50 μM)*. After allowing induction of AP for 30 min, the periplasmic, cytoplasmic and membrane fractions were isolated from equal number of each of the CCCP-treated and the control cells and the western blotting experiment was subsequently performed using anti-AP antibody. Lanes (a, b, c) and (e, f, g) represent the membrane, periplasmic and cytoplasmic fractions of control and CCCP-treated cells respectively; lane d represents purified AP.

Besides induction of hsps, protonophores were known to inhibit translocation of the membrane and periplasmic proteins, resulting in their accumulation in cell cytosol as non-functional precursor form [28-30]. In order to find out the detailed molecular correlation between protonophores-mediated induction of heat-shock-like response and inhibition of protein translocation, the inducible periplasmic protein AP of *E. coli *was selected here as the representative target protein for the translocation experiments. AP was a nonspecific phosphomonoesterase, used to generate inorganic phosphate from a variety of phosphorylated derivatives. The AP gene was known to be inducible as its expression was negatively regulated by the inorganic phosphate – the end product of AP digestion. Thus, the addition of phosphate to the growth medium repressed the induction of AP or in other words, phosphate-less growth medium induced AP in *E. coli *[31]. When AP was induced in presence of the protonophores, the level of cellular active AP, at any instant of growth, had decreased gradually by the presence of increasing concentrations of CCCP (0 – 50 μM) [fig. 4A] or DNP (0 – 1.5 mM) [not shown] in the growth medium. At 50 μM CCCP concentration, the amount of enzymatically active AP was almost absent. However, the western blot study of the periplasmic, cytoplasmic and membrane fractions of cells using anti-AP antibody (fig. 4B) showed that the lane g, where the cytoplasmic fraction of the CCCP-treated cells was loaded, had contained the induced AP. No considerable AP band was observed in the lanes (f & e), where the periplasmic and membrane fractions of the CCCP-treated cells were loaded respectively. On the other hand, in the case of CCCP-untreated control cells, approximately equal amount of AP was found to be present in both periplasmic (lane b) and cytoplasmic (lane c) fractions; no trace of AP was found in the membrane fraction (lane a). The AP in the cytoplasmic fraction of the control cells (lane c), perhaps, represented the amount of AP that had yet to be translocated to the periplasm. The result of this study revealed that by the presence of CCCP (50 μM) in the growth medium, the induced AP could not be transported out from the cytoplasm to the periplasm. The less intensity of the AP band in lane g compared to the sum of the intensities in lanes b and c implied less induction of AP in cells grown in the presence of CCCP with respect to the control cells; this was consistent with the fact of low growth rate of the CCCP-treated cells (result not shown). The result of this study, therefore, indicated that the presence of CCCP-like protonophore in the growth medium had partially reduced the induction of AP; however, the induced AP could not be transported out from the cytoplasm to the periplasm. AP was known to be synthesized initially in the cytoplasm and then translocated out through the inner membrane to be finally localized as dimeric, active form at the periplasm [32,33]. As the dimerization of AP, through the disulfide bond, could not take place in the reducing milieu of the cytoplasmic environment, the cytosolic pool of the nontranslocated AP in the CCCP-treated cells had shown no activity [34,35].

**Figure 5:**
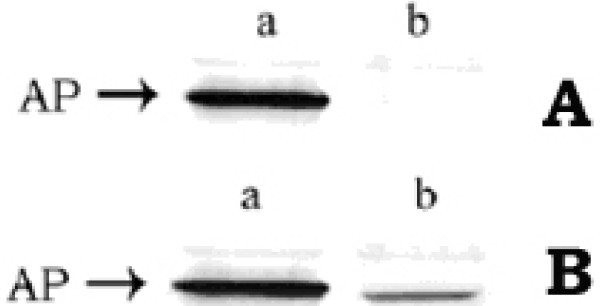
***The fate of translocation of cytosolic AP in E. coli MPh42 cells, after removal of CCCP***. A and B represent the autoradiograph and the western blot respectively. Lanes a and b represent the periplasmic fractions of the control and CCCP-treated cells respectively.

To investigate whether the non-translocated AP in cell cytosol could have been transported out to the periplasm on withdrawal of CCCP from the growth medium, pulse-chase and immunoprecipitation experiment was performed. Cells, grown in phosphate-free (required for the induction of AP) and methionine-free MOPS medium in the presence of 50 μM CCCP, were radio-labeled with ^35^S-methionine for 30 min; the CCCP was then removed by centrifugation and the cells were resuspended in the phosphate-less MOPS medium. Finally the chasing with cold methionine was allowed for 1 hr. The periplasmic fractions of the chased cells were isolated, immunoprecipitated with anti-AP antibody, the immunoprecipitates were run in 12% SDS-polyacrylamide gel, western blotting with anti-AP antibody was done and the blotted membrane was finally autoradiographed [36]. The autoradiograph (Fig. 5A) showed that the periplasmic fraction of the CCCP-treated cells had contained no trace of AP (lane b), whereas that of the control cells contained it (lane a). This signified that the AP, synthesized during the presence of CCCP (i.e., for the labeling period of 30 min), could not be translocated out to the periplasm, even after 60 min of chasing in the absence of CCCP. The western blot result (Fig. 5B) showed that the periplasmic fraction of both the CCCP-treated (lane b) and untreated (lane a) cells had contained AP. This implied that after the removal of CCCP, the newly synthesized AP (during the chase period of 60 min) had been exported out to the periplasm. This result can, therefore, be summarized as – the AP, once induced in the presence of CCCP and accumulated in the cell cytoplasm, had never crossed the cytoplasmic membrane (fig. 5A); on contrary the AP, newly induced in the same cells after withdrawal of CCCP, had crossed the cytoplasmic membrane to be located in the periplasm (Fig. 5B).

**Figure 6:**
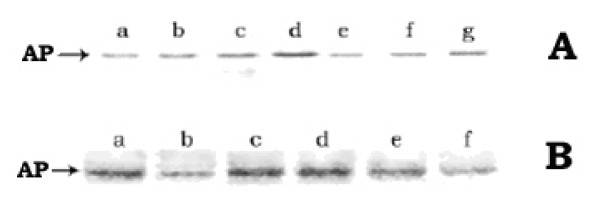
**A. W*estern blot of the soluble and insoluble fractions of the CCCP-treated E. coli MPh42 cells***. Cells were initially grown up to [OD]_600 nm _≈ 0.5 at 30°C in complete MOPS medium and were subsequently transferred to phosphate-less MOPS medium. They were then further allowed to grow at 30°C in the presence of 50 μM CCCP. At different instants of growth, the soluble and insoluble cell fractions were isolated as described in 'Methods' section. Lanes a, b, c represent the soluble and lanes e, f, g represent the insoluble fractions, isolated at 30, 60 and 90 min of growth respectively. Lane d represents purified AP. B. *Degradation of AP-aggregates in CCCP-treated cells, after removal of CCCP*. Lanes (a, b), (c, d) and (e, f) represent 0 hr and 3 hr of chasing for the strains SG20250, SG22159 and JT4000 respectively.

In order to investigate that whether any aggregation occurred in the non-functional, permanently stored AP pool in cell cytosol, the total soluble and insoluble fractions of cells were isolated at different intervals of growth in the presence of 50 μM CCCP, and the western blot study of the fractions was performed using anti-AP antibody. Both the fractions were found to contain AP (Fig. 6A), indicating that the stored AP was partly in the aggregated and partly in the dispersed form. Moreover, Fig. 6A showed that the amount of AP in each fraction had increased gradually with the time of AP induction in the presence of CCCP. It should be mentioned here that in the control cells, the amount of insoluble fraction was negligible and the AP was found to be present only in the soluble fraction (data not shown).

The presence of aggregated proteins in cells was reported to elicit induction of hsps for cell survival [17]. Therefore, in the following experiments, focus was made on the ultimate fate of the AP-aggregates in cytoplasm of the protonophores-treated cells, with a view to observe the role of induced hsps on the aggregates. The result of the following 'pulse-chase and immunoprecipitation' experiment on the *E. coli *strain SG20250 showed degradation of the AP-aggregate with time. For this study, log phase grown cells, in phosphate- and methionine-free MOPS medium, were allowed to label with ^35^S-methionine (30 μCi/ml) at 30°C in the presence of 50 μM CCCP. After 30 min of labeling, cells were transferred to fresh phosphate-free MOPS medium containing 1 mM BSA and 150 μg/ml spectinomycin and allowed to incubate further for 3 hr without CCCP. At 0 and 3 hr of chasing, equal volume of cell aliquot was withdrawn on ice, centrifuged and subjected to isolation of aggregated proteins. The isolated aggregates were immunoprecipitated with anti-AP antibody. The immunocomplex was run on 12% SDS-polyacrylamide gel, the gel was dried and subsequently set to autoradiography. The autoradiograph (Fig. 6B) of the electrophoresed immunoprecipitates indicated that the amount of AP-aggregate, after 3 hr of chasing (lane b), was about 66% less than its initial amount at 0 hr of chasing (lane a). This signified that the AP-aggregate had been degraded finally with time. It seemed that the degradation of AP-aggregate had been possibly caused by some induced heat-shock protease(s). When the degradation of the CCCP-mediated AP-aggregate was checked, by the same 'pulse-chase and immunoprecipitation' experiment in two different *E. coli *mutants for the heat-shock proteases Lon (JT4000) and ClpP (SG22159), it was observed that in the c*lpP *mutant, no degradation of the AP-aggregate took place (lanes c and d, Fig. 6B); whereas in the *lon *mutant, degradation occurred (lanes e and f, Fig. 6B). This result clearly implied that not the major heat-shock protease Lon, rather a minor protease ClpP was responsible for the degradation phenomenon. Such degradation removed the translocation-incompetent, non-functional AP and thus was essential for cell survival; this was supplemented from the fact that the *clpP *mutant (SG22159) was more sensitive to CCCP than wild type strain SG20250. In the presence of 25 μM CCCP, where the wild type cells had some growth, the mutant cells became bacteriostatic, and by the treatment of 50 μM CCCP for 90 min, where there was no killing of *E. coli *SG20250 cells, about 90% cell-killing occurred in case of *E. coli *SG22159 strain (data not shown).

**Figure 7:**
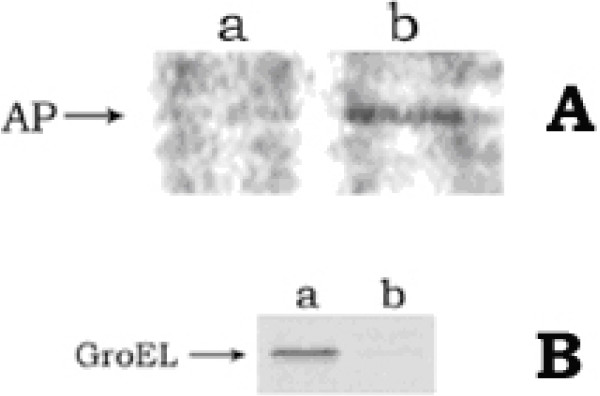
**A. *Formation of AP-DnaK binary complex in CCCP-treated cells***. Log phase cells, in phosphate-free MOPS medium, were labeled with ^35^S-methionine (30 μCi/ml) for 30 min at 30°C in presence of 50 μM CCCP. 1 ml labeled cells was chilled, centrifuged and resuspended in 200 μl Tris buffer (30 mM, pH 8.0) containing 20% sucrose, 10 mM EDTA (pH 8.0), 1 mg/ml lysozyme and the cell suspension was kept at 4°C for 10 min. 1 ml lysis solution [50 mM Tris (pH 8.0), 40 mM NaCl and 0.1% Tween 20] was added to the cell suspension and placed on ice for 30 min; NaCl was then added to a final concentration of 0.2 M and the cell lysate was centrifuged at 10,000 rpm for 10 min at 4°C. The supernatant was first immunoprecipitated with anti-DnaK antibody. The immunocomplex was washed with above lysis solution containing 0.2 M NaCl, suspended in 100 μl Tris (pH 7.4), heated at 100°C for 3 min and finally immunoprecipitated with anti-AP antibody. The immunoprecipitate was run in 12% SDS-polyacrylamide gel and finally phosphorimaged. Lane a: CCCP-treated cell; lane b: control cell. B. *State of GroEL induction in cells containing excess DnaK*. Transformed cells were primarily grown up to log phase (~1.5 × 10^8 ^cells/ml) at 30°C in MOPS medium. 1 mM IPTG was then added and growth was allowed for another 30 min (to induce DnaK). The cells were transferred to methionine-free MOPS medium, grown further in presence of 50 μM CCCP for 20 min and then labeled with ^35^S-metthionine (30 μCi/ml) for 10 min. Parallel experiment was done for untransformed cells also. Cell extracts were then prepared by boiling with SDBME buffer. Equal amount of protein extract from both transformed and untransformed cells, as estimated by Bradford method, was subjected to immunoprecipitation using anti-GroEL antibody. The immunoprecipitate was run in SDS-polyacylamide gel and phosphoroimaged. Lane a: untransformed cell; lane b: transformed cell.

When the cell extract of AP-induced culture was subjected to two-step immunoprecipitation study using anti-DnaK and anti-AP antibodies serially, the final immunoprecipitate of the CCCP-treated cells, in contrast to that of the control cells, had contained AP in addition to the DnaK protein (fig. 7A). This clearly signified that the first immunoprecipitate with anti-DnaK antibody had certainly contained AP i.e., the non-translocated AP in the CCCP-treated cells was present in cell cytosol as a binary complex form with DnaK. This result justified the fact of sigma-32 stabilization in the protonophores-treated cells as – the non-translocated proteins had signaled DnaK/J to bind with them, finally freeing and so stabilizing sigma-32. This was further confirmed by our observation that in the cells containing over-expressed DnaK, the CCCP could not trigger the induction of hsps (fig. 7B); this was because the sigma-32 could not be freed from the DnaK to bind with the RNA polymarease, due to the excess cellular pool of DnaK protein. For this study, cells of *E. coli *MPh42 were transformed with plasmid pET vector containing *dnaK *gene and the DnaK protein was over-expressed by using 1 mM IPTG in the MOPS growth medium. When such excess DnaK-containing cells were subsequently grown in the presence of 50 μM CCCP and the cell extract was immunoprecipitated using anti-GroEL antibody, no induction of GroEL had been observed in the CCCP-treated transformed cells (lane b, fig. 7B); whereas the induction had occurred in the CCCP-treated untransformed cells (lane a, fig. 7B). This result implied that no induction of hsps had taken place in the CCCP-treated cells having excess amount of DnaK chaperone.

## Conclusion

The whole study can, therefore, be concluded as: the protonophores like CCCP and DNP, by blocking the translocation of membrane and periplasmic proteins in *E. coli*, caused cytoplasmic accumulation of those proteins – partly in the insoluble aggregated form and partly in the soluble dispersed form; such stored proteins, which could never be translocated out even after removal of the protonophores, had induced cellular heat-shock response enhancing the syntheses of a few heat-shock chaperones and proteases – perhaps, the heat-shock protease ClpP ultimately degraded the non-translocated protein-aggregates to remove them from the cell. Moreover, the induction of hsps had taken place mainly due to stabilization of the normally unstable heat-shock regulator protein sigma-32; the stabilization had occurred due to titration of the chaperone system DnaK/J by the non-translocated, inactive periplasmic and membrane proteins stored in the cytoplasm of the CCCP-treated cells, because the titration consequently made the sigma-32 free of DnaK/J and so prevented its cleavage by the FtsH protease.

## Authors' contributions

BJ contributed substantially in designing experiments and in acquisition, analysis and interpretation of data. SP and SS contributed physically and intellectually during experimentations. TB contributed by conceptualizing the original problem, discussing the results time to time and finally preparing the manuscript. All authors read and approved the final manuscript.
